# Supplementary material for: Multi-species, multi-transcription factor binding highlights conserved control of tissue-specific biological pathways
Source: eLife. 2014 Oct 3;3:e02626. doi: 10.7554/eLife.02626 (PMC4359374; doi:10.7554/eLife.02626)
Supplement: Figure 1—source data 1. — DOI: http://dx.doi.org/10.7554/eLife.02626.004 [file elife02626s001.docx]

Table of Contents

Figure 1-source data 1A. Analysis and quality control of ChIP-seq experiments – part 1. 1

Figure 1-source data 1B. Analysis and quality control of ChIP-seq experiments – part 2. 4

Figure 1-source data 1C. Analysis and quality control of ChIP-seq experiments – part 3. 6

Figure 1-source data 1D. Epitope regions from antibodies used in this study. 7

Figure 1-source data 1E. Comparison of ChIP-seq peak parameters on CRM building. 8

Figure 1-source data 1F. Pairwise Analysis of CRM conservation yields similar percent conservation using different multiple sequence alignments. 9

Figure 1-source data 1G. Effect of changing the required base-pair overlap within the multiple sequence alignments on the number of shared human CRMs. 9

## Figure 1-source data 1A. Analysis and quality control of ChIP-seq experiments – part 1.

The ENCODE consortia have developed several quality metrics to help evaluate the quality of ChIP-seq experiments ([Landt et al. 2012](#_ENREF_1)). We used these metrics to describe the quality of our data. FRiP: Fraction of reads that lie in detected peak regions using MACs with an FDR threshold of 0.01. ENCODE guidelines suggest data sets with a FRiP <1% should be reviewed. PBC: PCR Bottleneck Coefficient. This measure gives a library complexity estimate. PBC<0.5 indicates PCR bottlenecks, 10% of ENCODE libraries show this level of PBC. Two of the libraries used to define peaks in our analysis had PBC values of less than 0.5 (human (HH1294) FOXA1 and dog (cfa4) FOXA1; labelled in pink). We retained these libraries in our CRM analyses as both of these libraries passed other QC metrics, gave peaks with a central FOXA1 motif, and came from a valuable biological specimen for which other TF experiments passed all QC metrics. We also used ENCODE’s cross-correlation analysis, which is a peak calling-independent measurement of ChIP-seq quality. Landt et al suggest that libraries with NSC below 1.05 and RSC below 0.8 may be of low quality and recommend an additional replicate be attempted. All our samples used in our comparative CRM analysis passed this criteria. Table S1 shows: Species, Replicate ID, Factor that was pulled down, Number of uniquely mapped reads in the library to the reference genome (see main Materials and Methods), Number of peaks called with SWEMBL parameter R of 0.005, Number of peaks called with MACS2 (used for ENCODE analyses only) parameter q (FDR) 0.01, FRiP, PBC, NSC, RSC.

| Species | Replicate | Factor | # Uniq mapped reads | SWEMBL R 0.005 | MACS FDR 0.01 | FRiP | PBC | NSC | RSC |
| --- | --- | --- | --- | --- | --- | --- | --- | --- | --- |
| **Cfam** | cfa3 | CEBPa | 19249572 | 52523 | 34529 | 0.1362 | 0.92 | 1.83 | 1.81 |
|  | cfa4 | CEBPa | 16562323 | 64899 | 70358 | 0.3046 | 0.91 | 3.2 | 1.88 |
|  | cfa3 | FoxA1 | 14043705 | 30764 | 5518 | 0.0152 | 0.98 | 1.12 | 1.06 |
|  | cfa4 | FoxA1 | 16406575 | 63091 | 34968 | 0.2973 | 0.31 | 3.96 | 2.35 |
|  | cfa3 | HNF4a | 19928953 | 64649 | 60987 | 0.5201 | 0.79 | 5.72 | 1.98 |
|  | cfa4 | HNF4a | 17704928 | 48613 | 49833 | 0.2801 | 0.76 | 3 | 2.12 |
|  | cfa3 | ONECUT1 | 23778441 | 21141 | 13551 | 0.0501 | 0.7 | 1.41 | 3.04 |
|  | cfa4* | ONECUT1 | 19144417 | 10931 | 5483 | 0.0135 | 0.76 | 1.21 | 2.98 |
| **Hsap** | hsaHH1294 | CEBPa | 19114189 | 40270 | 34880 | 0.0997 | 0.74 | 1.62 | 2.33 |
|  | hsaHH1323 | CEBPa | 18177941 | 48259 | 20887 | 0.0982 | 0.81 | 1.97 | 2.02 |
|  | hsaCRI3 | FoxA1 | 14664082 | 42538 | 29290 | 0.0884 | 0.81 | 1.67 | 1.7 |
|  | hsaHH1294 | FoxA1 | 12870588 | 130108 | 48722 | 0.4152 | 0.14 | 5.82 | 2.04 |
|  | hsaHH1294 | HNF4a | 15425128 | 66498 | 64285 | 0.2438 | 0.88 | 2.66 | 1.67 |
|  | hsaHH1308 | HNF4a | 13966010 | 51974 | 22468 | 0.1164 | 0.94 | 1.99 | 1.84 |
|  | hsaHH1294 | ONECUT1 | 14495940 | 32111 | 25316 | 0.0894 | 0.81 | 1.79 | 1.9 |
|  | hsaHH1328* | ONECUT1 | 17116428 | 22518 | 15440 | 0.0732 | 0.94 | 1.65 | 1.56 |
| **Mmul** | mml138** | CEBPa | 13095535 | 76578 | 72850 | 0.2362 | 0.97 | 2.81 | 1.82 |
|  | mmlBlues | CEBPa | 24160837 | 22286 | 18263 | 0.0411 | 0.77 | 1.26 | 1.76 |
|  | mmlBob* | CEBPa | 20592695 | 21549 | 20477 | 0.0395 | 0.84 | 1.18 | 1.39 |
|  | mmlJosie** | CEBPa | 26715026 | 23656 | 32126 | 0.0659 | 0.92 | 1.29 | 1.47 |
|  | mml138** | FoxA1 | 11565170 | 54844 | 9235 | 0.0245 | 0.86 | 1.34 | 1.34 |
|  | mmlBlues | FoxA1 | 15340944 | 62468 | 45717 | 0.1899 | 0.83 | 2.31 | 1.85 |
|  | mmlBob | FoxA1 | 22663226 | 31345 | 32321 | 0.0859 | 0.5 | 1.58 | 2.08 |
|  | mml138** | HNF4a | 15367667 | 26703 | 24311 | 0.0866 | 0.94 | 1.56 | 1.47 |
|  | mmlBlues | HNF4a | 27523197 | 40378 | 33782 | 0.1383 | 0.94 | 1.68 | 1.41 |
|  | mmlBob* | HNF4a | 18515864 | 12894 | 10415 | 0.0207 | 0.93 | 1.09 | 0.89 |
|  | mmlJosie** | HNF4a | 24114102 | 18526 | 24131 | 0.0636 | 0.94 | 1.35 | 1.38 |
|  | mmlBlues | ONECUT1 | 11846247 | 104506 | 13990 | 0.07 | 0.47 | 2.1 | 2.69 |
|  | mmlBob* | ONECUT1 | 18548881 | 14711 | 11052 | 0.0662 | 0.1 | 2.59 | 2.85 |
| **Mmus** | BL60ON562 | CEBPa | 18139873 | 53242 | 58322 | 0.2248 | 0.94 | 2.47 | 1.81 |
|  | OON489 | CEBPa | 6906209 | 69269 | 44025 | 0.2768 | 0.9 | 3.9 | 1.62 |
|  | OON404 | FoxA1 | 15283711 | 75467 | 60763 | 0.3498 | 0.85 | 3.54 | 1.65 |
|  | OON405 | FoxA1 | 15410267 | 52639 | 44098 | 0.1562 | 0.81 | 1.91 | 1.91 |
|  | 0h490+491 | HNF4a | 19954380 | 77726 | 94844 | 0.5854 | 0.83 | 5.13 | 1.79 |
|  | OON489 | HNF4a | 15407264 | 87886 | 91128 | 0.6462 | 0.81 | 7.01 | 1.37 |
|  | 0h490+491 | ONECUT1 | 21057518 | 54259 | 64811 | 0.3177 | 0.65 | 3.68 | 2.03 |
|  | mmu12 | ONECUT1 | 22094371 | 52946 | 43837 | 0.2187 | 0.9 | 2.6 | 1.92 |
| **Rnor** | rno5 | CEBPa | 12823235 | 71976 | 61257 | 0.3351 | 0.84 | 3.7 | 1.65 |
|  | rno7 | CEBPa | 20424123 | 34259 | 37603 | 0.1325 | 0.9 | 1.72 | 1.86 |
|  | rno5 | FoxA1 | 11489479 | 81934 | 65137 | 0.3912 | 0.82 | 4.66 | 1.79 |
|  | rno8 | FoxA1 | 17093789 | 45583 | 46612 | 0.2052 | 0.93 | 2.31 | 1.77 |
|  | rno5 | HNF4a | 10424552 | 94678 | 55536 | 0.3775 | 0.75 | 4.39 | 1.47 |
|  | rno7 | HNF4a | 19941373 | 33439 | 35427 | 0.1197 | 0.9 | 1.54 | 1.65 |
|  | rno5 | ONECUT1 | 10420671 | 59080 | 35978 | 0.195 | 0.84 | 2.99 | 2.03 |
|  | rno8 | ONECUT1 | 22246176 | 21866 | 21990 | 0.0791 | 0.93 | 1.46 | 1.78 |

*Replicates used for validation only

** Replicates not used for further analysis.

##

## Figure 1-source data 1B. Analysis and quality control of ChIP-seq experiments – part 2.

To assess the correlation between replicates RPKM values of each ChIP-seq samples, relative to input DNA, were calculated with DiffBind Bioconductor package ([Stark 2011](#_ENREF_4)). Counts are reflective of Pearson correlation values between sample pairs. Note that we used SWEMBL peaks for the DiffBind analysis. As a second, independent measurement of quality we also performed the Irreproducible Discovery Rate (IDR) analysis describe by ENCODE ([Landt et al. 2012](#_ENREF_1)). IDR was also performed for each pair of replicate ChIP-seq samples as described (https://sites.google.com/site/anshulkundaje/projects/idr)([Landt et al. 2012](#_ENREF_1)). IDR is calculated between the peaks discovered in: replicates (Nt), pooled and randomly separated replicates (pooled pseudo-replicates, Np), and randomly separated replicates (self pseudo-replicates, N1 and N2). MACS2 ([Zhang et al. 2008](#_ENREF_7)) (https://github.com/taoliu/MACS/) was used to call peaks for IDR. Peaks were ranked by p-value, and the top 100000 were compared between sets of replicates or pseudo-replicates to obtain IDR. ENCODE recommends Nt/Np and N1/N2 ratios within a factor of 2 (> 0.5 and < 2) in order to demonstrate reproducibility between replicates. Five replicates were used for validation purposes only (**) as they gave less peaks and lower signal to noise ratio. The human ONECUT1 replicate hsaHH1328 was from female liver and used only for validation proposes as all other samples compared in this study were from males.

| **Species** | **Factor** | **Replicate 1** | **Replicate 2** | **Correlation** | **Nt / Np** | **N1 / N2** |
| --- | --- | --- | --- | --- | --- | --- |
| **Hsap** | CEBPA | hsaHH1294 | hsa1323 | 0.79 | 0.73 | 0.91 |
|  | HNF4A | hsaHH1294 | hsaHH1308 | 0.73 | 0.66 | 1.51 |
|  | FOXA1 | hsaHH1294 | hsaCRI3 | 0.74 | 0.64 | 3.09 |
|  | ONECUT1 | hsaHH1294 | hsaHH1328** | 0.77 | 0.77 | 1.01 |
| **Mmul** | CEBPA | mmlBlues | mmlBob** | 0.51 | 0.88 | 1.20 |
|  | HNF4A | mmlBlues | mmlBob** | 0.53 | 0.50 | 2.65 |
|  | FOXA1 | mmlBlues | mmlBob | 0.80 | 0.60 | 0.97 |
|  | ONECUT1 | mmlBlues | mmlBob** | 0.73 | 0.40 | 0.43 |
| **Mmus** | CEBPA | OON489 | BL60ON562 | 0.81 | 1.10 | 0.90 |
|  | HNF4A | OON489 | 0h490+491 | 0.97 | 1.06 | 0.99 |
|  | FOXA1 | OON404 | OON405 | 0.87 | 0.90 | 1.34 |
|  | ONECUT1 | mmu12 | 0h490+491 | 0.96 | 0.99 | 0.97 |
| **Rnor** | CEBPA | rno5 | rno7 | 0.94 | 0.92 | 1.43 |
|  | HNF4A | rno5 | rno7 | 0.92 | 0.86 | 1.57 |
|  | FOXA1 | rno5 | rnor8 | 0.91 | 0.98 | 1.27 |
|  | ONECUT1 | rno5 | rno8 | 0.86 | 1.07 | 1.37 |
| **Cfam** | CEBPA | cfa3 | cfa4 | 0.96 | 0.97 | 1.22 |
|  | HNF4A | cfa3 | cfa4 | 0.94 | 0.89 | 0.73 |
|  | FOXA1 | cfa3 | cfa4 | 0.85 | 0.83 | 2.91 |
|  | ONECUT1 | cfa3 | cfa4** | 0.85 | 0.54 | 0.35 |

** Replicate used for validation only

## Figure 1-source data 1C. Analysis and quality control of ChIP-seq experiments – part 3.

RPKM values of replicate ChIP-seq samples, relative to input DNA, were calculated with DiffBind Bioconductor package ([Stark 2011](#_ENREF_4)). Counts are reflective of Pearson correlation values between sample pairs. The heatmap was generated using unsupervised hierarchical clustering.


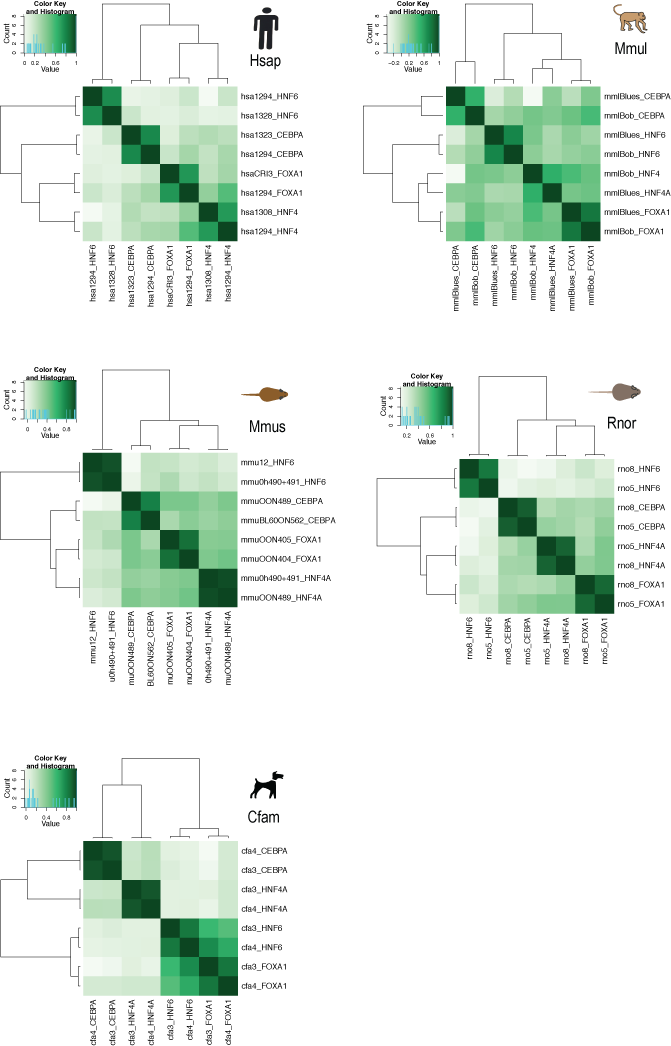


## Figure 1-source data 1D. Epitope regions from antibodies used in this study.

Multiple species sequence alignments of the protein regions from which the antibodies used in this study were raised. A) HNF4A antibody ARP31946 has previously been described for ChIP-seq in human, mouse and dog HNF4A (Schmidt et al. 2010). B) FOXA1 antibody ab5089 has been previously validated in mouse and human ChIP-seq studies ([Motallebipour et al. 2009](#_ENREF_2); [Stefflova et al. 2013](#_ENREF_5)). The dog FOXA1 coding region orthologous to the human immunizing peptide fell within a gap in the dog genome. We used PCR to amplify dog genomic DNA and filled this gap (deposited under GenBank accession number JN601139). C) Manufacturer released region of the human HNF6/ONECUT1 protein (aa 11-110) to which the rabbit anti-human polyclonal antibody sc13050 antibody was designed. This antibody has previously been validated in mouse and human studies ([Odom et al. 2007](#_ENREF_3); [Wilson et al. 2008](#_ENREF_6)). The macaque HNF6/ONECUT1 coding region orthologous to the human immunizing region fell within a gap in the macaque genome. We used PCR of macaque genomic DNA to fill this gap and deposited the sequence in GenBank under accession number JQ178331. D) The antibody goat anti-human polyclonal antibody sc-9314 has been shown to specifically recognize the C-terminus of CEBPA.


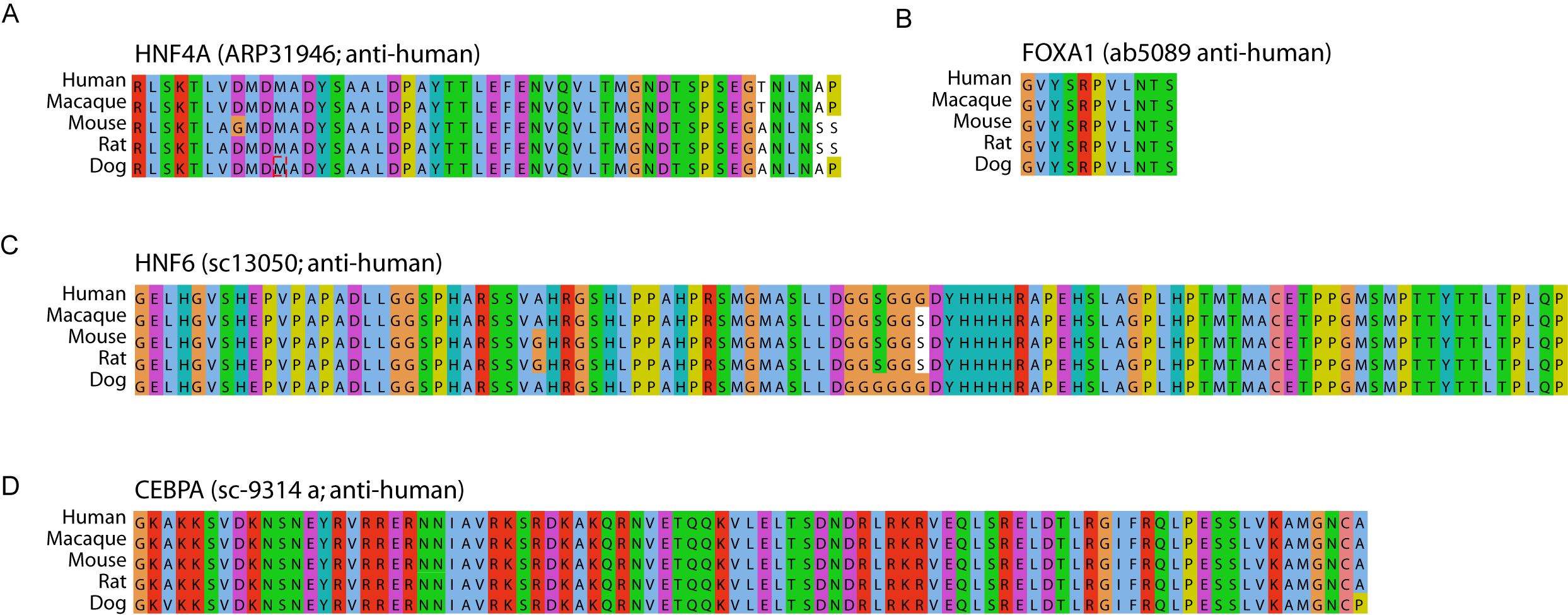


## Figure 1-source data 1E. Comparison of ChIP-seq peak parameters on CRM building.

We asked how building human CRMs with individual biological replicates or if using a different peak caller (MACS) affected the phylogenetic classification of categorized CRM/Singleton data used in this study. The “Best” data refers to the choice of human replicates used for this study. When CRMs or singletons overlapped two events in our “best data” (< 2.0% of events), it was removed form the analysis to avoid arbitrarily selecting a category. The “Hi rep” refers to CRMs built using the replicate that gave the most peaks; “Lo rep” refers to the CRMs built using the replicate with the fewest peaks. MACs refers to the CRMs built with our “Best” data using the MACS peak caller at an FDR of 0.05.

| Category | Best data  (this study) | Replicate  highest peak # (Hi) | | Replicate with  lowest peak # (Lo) | | MACs Peak   on "Best" data (FDR 0.05) | |
| --- | --- | --- | --- | --- | --- | --- | --- |
|  |  | hi CRM | hi singleton | lo CRM | lo singleton | macs CRM | macs Singleton |
| human_only_CRM | 19462 | 18321 | 604 | 12716 | 5566 | 18756 | 524 |
| primate_shared_CRM | 4672 | 4404 | 126 | 3850 | 687 | 4527 | 85 |
| beyond_primate_CRM | 7631 | 7048 | 265 | 5888 | 1455 | 7303 | 219 |
| human_only_singleton | 30718 | 10397 | 17489 | 2452 | 16094 | 11025 | 16562 |
| primate_shared_singleton | 6125 | 2200 | 3505 | 627 | 3967 | 2327 | 3457 |
| beyond_primate_singleton | 6981 | 2820 | 3595 | 1007 | 4314 | 3037 | 3489 |
| CRM/singleton not in "Best" | 0 | 5247 | 56204 | 1452 | 18172 | 7947 | 46069 |
| total in "Best" | 75589 | 45190 | 25584 | 26540 | 32083 | 46975 | 24336 |
| total CRM "Best" | 31765 | 29773 | 995 | 22454 | 7708 | 30586 | 828 |
| total singleton "Best" | 43824 | 15417 | 24589 | 4086 | 24375 | 16389 | 23508 |
| total CRMS/singletons | 75589 | 50437 | 81788 | 27992 | 50255 | 54922 | 32283 |

##
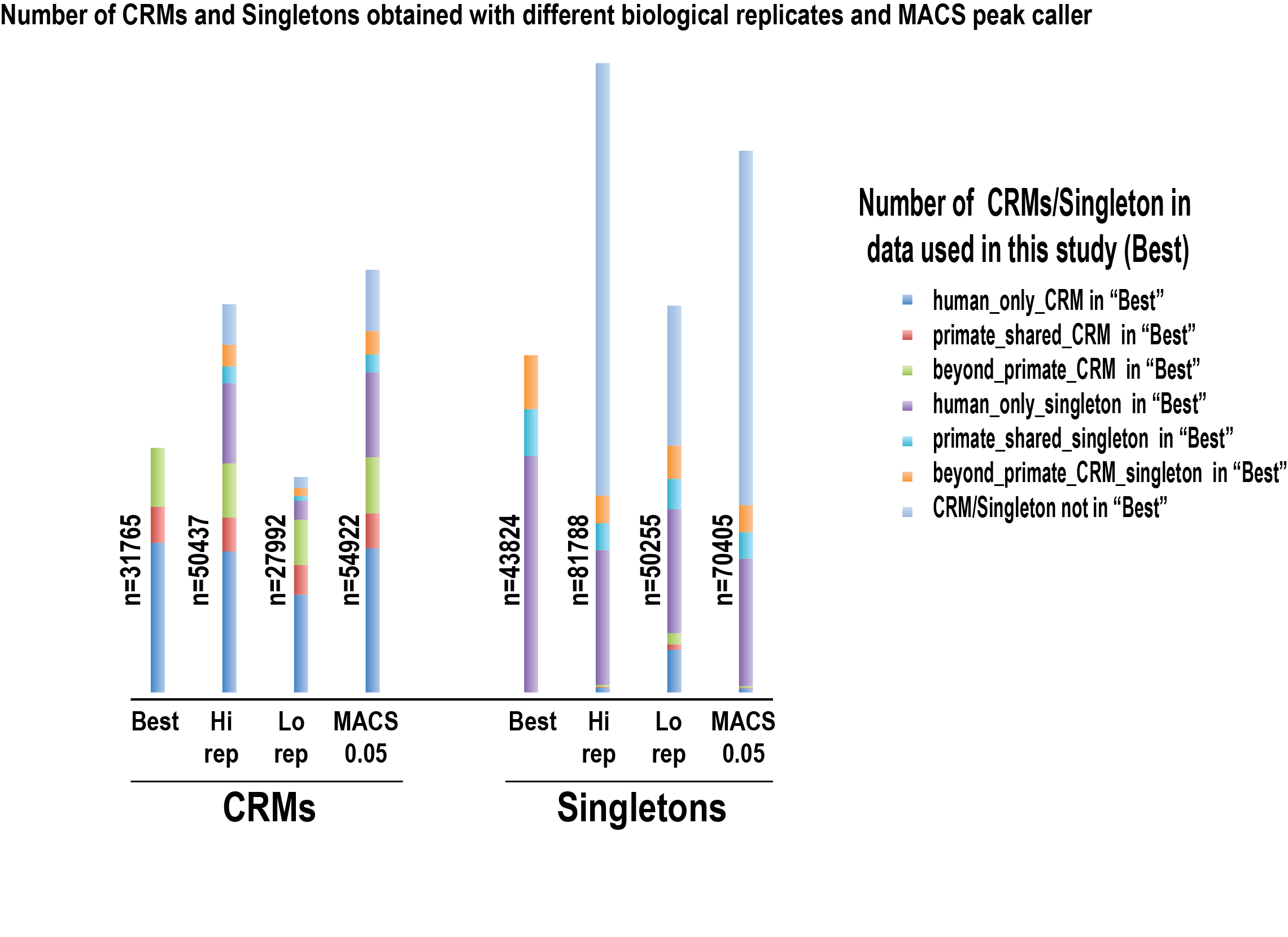


## Figure 1-source data 1F. Pairwise Analysis of CRM conservation yields similar percent conservation using different multiple sequence alignments.

Shared binding events were initially identified in a pair-wise fashion using the 9-way EPO-MSA and 12-way PECAN-MSA.

| **CRMs** | **TFs in EPO** | **Dog** | **Rat** | **Mouse** | **Macaque** | **Human** | **TFs in PECAN** |
| --- | --- | --- | --- | --- | --- | --- | --- |
| **Human** | 31,157 | 4,626  16% | 3,892  12% | 5,293  14% | 8,818  35% |  | 22,840 |
| **Macaque** | 19,631 | 3,482  15% | 2,994  11% | 4,069  12% |  | 6,235  34% | 13,751 |
| **Mouse** | 46,417 | 4,407  12% | 12,587  32% |  | 3,073  13% | 4,153  15% | 34,111 |
| **Rat** | 32,916 | 3,438  11% |  | 9,340  32% | 2,278  12% | 2,978  13% | 23,519 |
| **Dog** | 27,485 |  | 2,447  12% | 3,289  13% | 2,216  14% | 3,078  15% | 17,571 |

##

## Figure 1-source data 1G. Effect of changing the required base-pair overlap within the multiple sequence alignments on the number of shared human CRMs.

CRMs in a second species were defined as shared with human if: 1) they fell near 50bp (+50ext) of an orthologous position in the MSA; 2) overlapped by 1 bp; 3) overlapped by 10bp (parameter used in this study); 4) overlapped by 25 bp; or 5) overlapped by 50 bp.

|  | **% human CRMs shared with a second species**. | | | |
| --- | --- | --- | --- | --- |
| **Overlap** | **Mmul** | **Mmus** | **Rnor** | **Cfam** |
| **+50ext(*)** | 34.7 | 13.6 | 12.1 | 15.8 |
| **1bp** | 33.5 | 12.0 | 10.7 | 14.4 |
| **10bp** | 33.6 | 12.1 | 10.8 | 14.6 |
| **25bp** | 33.2 | 11.8 | 10.5 | 14.2 |
| **50bp** | 32.8 | 11.4 | 10.2 | 13.9 |
| **Average** | 33.6 | 12.2 | 10.9 | 14.6 |

|  | **% human CRMs with all 4 TFs shared with a second species** | | | |
| --- | --- | --- | --- | --- |
| **Overlap** | Mmul | Mmus | Rnor | Cfam |
| **+50ext** | 47.9 | 22.7 | 18.0 | 22.9 |
| **1bp** | 46.3 | 19.9 | 16.1 | 21.3 |
| **10bp** | 46.1 | 19.8 | 15.9 | 21.3 |
| **25bp** | 45.8 | 19.5 | 15.6 | 21.1 |
| 50bp | 45.2 | 19.0 | 15.4 | 20.8 |
| Average | 46.3 | 20.2 | 16.2 | 21.5 |

**References**

Landt SG, Marinov GK, Kundaje A, Kheradpour P, Pauli F, Batzoglou S, Bernstein BE, Bickel P, Brown JB, Cayting P et al. 2012. ChIP-seq guidelines and practices of the ENCODE and modENCODE consortia. *Genome Res* **22**(9): 1813-1831.

Motallebipour M, Ameur A, Reddy Bysani MS, Patra K, Wallerman O, Mangion J, Barker MA, McKernan KJ, Komorowski J, Wadelius C. 2009. Differential binding and co-binding pattern of FOXA1 and FOXA3 and their relation to H3K4me3 in HepG2 cells revealed by ChIP-seq. *Genome Biol* **10**(11): R129.

Odom DT, Dowell RD, Jacobsen ES, Gordon W, Danford TW, MacIsaac KD, Rolfe PA, Conboy CM, Gifford DK, Fraenkel E. 2007. Tissue-specific transcriptional regulation has diverged significantly between human and mouse. *Nat Genet* **39**(6): 730-732.

Stark RB, G.D. 2011. DiffBind: differential binding analysis of ChIP-Seq peak data. In *R package version 100*.

Stefflova K, Thybert D, Wilson MD, Streeter I, Aleksic J, Karagianni P, Brazma A, Adams DJ, Talianidis I, Marioni JC et al. 2013. Cooperativity and rapid evolution of cobound transcription factors in closely related mammals. *Cell* **154**(3): 530-540.

Wilson MD, Barbosa-Morais NL, Schmidt D, Conboy CM, Vanes L, Tybulewicz VL, Fisher EM, Tavare S, Odom DT. 2008. Species-specific transcription in mice carrying human chromosome 21. *Science* **322**(5900): 434-438.

Zhang Y, Liu T, Meyer CA, Eeckhoute J, Johnson DS, Bernstein BE, Nusbaum C, Myers RM, Brown M, Li W et al. 2008. Model-based analysis of ChIP-Seq (MACS). *Genome Biol* **9**(9): R137.
